# Antioxidant Therapy in Parkinson’s Disease: Insights from *Drosophila melanogaster*

**DOI:** 10.3390/antiox9010052

**Published:** 2020-01-07

**Authors:** Federica De Lazzari, Federica Sandrelli, Alexander J. Whitworth, Marco Bisaglia

**Affiliations:** 1 Department of Biology, University of Padova, 35131 Padova, Italy.federica.sandrelli@unipd.it (F.S.); 2 Medical Research Council Mitochondrial Biology Unit, University of Cambridge, Cambridge Biomedical Campus, Cambridge CB2 0XY, UK; a.whitworth@mrc-mbu.cam.ac.uk

**Keywords:** antioxidants, *Drosophila melanogaster*, oxidative damage, Parkinson’s disease, SOD-mimetics

## Abstract

Reactive oxygen species (ROS) play an important role as endogenous mediators in several cellular signalling pathways. However, at high concentrations they can also exert deleterious effects by reacting with many macromolecules including DNA, proteins and lipids. The precise balance between ROS production and their removal via numerous enzymatic and nonenzymatic molecules is of fundamental importance for cell survival. Accordingly, many neurodegenerative disorders, including Parkinson’s disease (PD), are associated with excessive levels of ROS, which induce oxidative damage. With the aim of coping with the progression of PD, antioxidant compounds are currently receiving increasing attention as potential co-adjuvant molecules in the treatment of these diseases, and many studies have been performed to evaluate the purported protective effects of several antioxidant molecules. In the present review, we present and discuss the relevance of the use of *Drosophila melanogaster* as an animal model with which to evaluate the therapeutic potential of natural and synthetic antioxidants. The conservation of most of the PD-related genes between humans and *D. melanogaster*, along with the animal’s rapid life cycle and the versatility of genetic tools, makes fruit flies an ideal experimental system for rapid screening of antioxidant-based treatments.

## 1. Introduction

**Figure 1:**
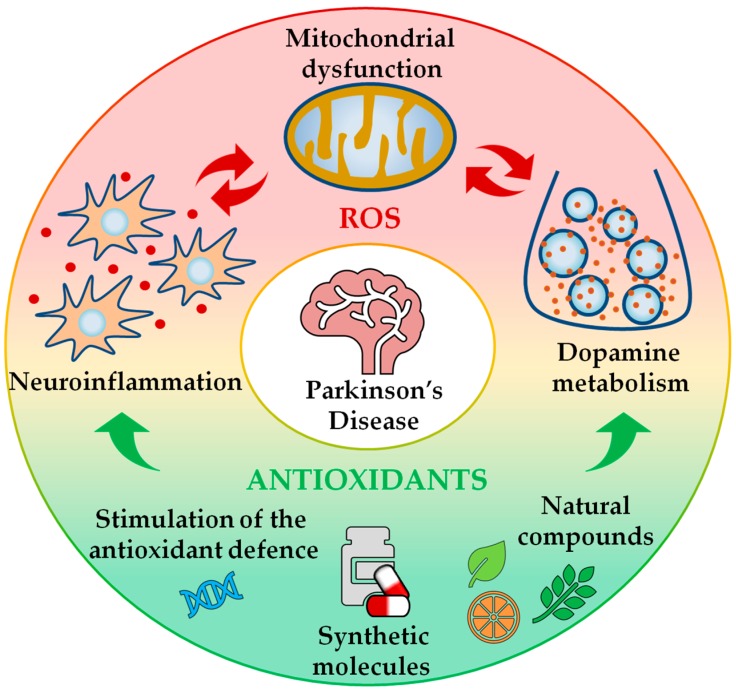
Beneficial effects of antioxidant treatment on the maintenance of redox homeostasis in Parkinson’s disease (PD). PD pathology is associated with an unbalanced redox state, which is the result of mitochondrial dysfunction, neuroinflammation and dopamine metabolism. Antioxidant therapies can help to hinder excessive oxidative stress conditions by buffering reactive oxygen species (ROS) production and limiting ROS-related damage. Antioxidant treatments encompass both natural (e.g., vitamins and plant extracts) and synthetic compounds (e.g., superoxide dismutase-mimetics), and can promote the stimulation of the endogenous antioxidant defence system. Therefore, the antioxidant treatment can act as a co-adjuvant to currently used PD therapies.

Parkinson’s disease (PD) is a chronic and progressive movement disorder that affects approximately 1–2% of the population over the age of 65 years. Clinically, the pathology is mainly characterised by resting tremor, rigidity, bradykinesia and postural instability. However, the symptomatology of the disease is nowadays considered more complex, also including non-motor symptoms such as depression, sleep disturbance and cognitive decline [1]. From a neuropathological point of view, PD is characterised by a preferential loss of dopaminergic neurons within the substantia nigra pars compacta, and by the deposition of intracellular inclusions, referred to as Lewy bodies (LBs), in the surviving neurons [2]. Although the majority of PD cases are idiopathic, the identification of genetic forms of the pathology has contributed to partially elucidating the cellular mechanisms behind the syndrome. Familial manifestations account for ~10% of the disease and are classified into dominant and recessive forms according to the pattern of inheritance [3]. Among the causative genes, *SNCA* and *LRRK2* are associated with dominant PD cases, while *Parkin*, *PINK1* and *DJ*-*1* contribute to recessive forms of the disease [4]. α-Synuclein is the major component of LBs in sporadic PD forms, while *LRRK2* is a kinase implicated in different cellular functions, including vesicle trafficking, synaptic morphogenesis and neurite outgrowth. *Parkin*, *PINK1* and *DJ-1* are all proteins differentially involved in mitochondrial homeostasis. *Parkin*, an E3 ubiquitin ligase, and *PINK1*, a serine–threonine protein kinase, cooperate to maintain mitochondrial quality control, driving the disposal of damaged or old mitochondria, while *DJ-1* is a multitasking protein principally implicated in the activation of antioxidant responses [4]. Although it is still unclear what triggers PD, growing bodies of evidence have suggested that unbalanced redox homeostasis is a common feature underlying both sporadic and idiopathic manifestations [5]. As previously discussed, multiple sources appear to contribute to the redox alterations observed in the disease, including mitochondrial dysfunction, neuroinflammation and dopamine metabolism [5]. Mitochondria have received special interest in PD etiopathology, especially considering that numerous PD-related genes and neurotoxins, such as MPTP, rotenone and paraquat (PQ), have been shown to affect mitochondrial functionality [6]. As mitochondria are considered a primary source of reactive oxygen species (ROS), mitochondrial dysfunctions are believed to abundantly participate in driving the state of oxidative stress observed in the disease [7]. Similarly, through the chronic activation of microglia, neuroinflammation can be responsible for a conspicuous production of ROS, which, if not detoxified, contributes to amplification of the state of oxidative stress [8]. Additionally, dopaminergic neurons are particularly vulnerable to oxidative damage because the metabolism of dopamine can itself act as a further source of ROS in the disease [9]. In light of these considerations, antioxidants are currently receiving attention as co-adjuvant molecules in PD treatment, and many studies in this frame have been published using different animal models of PD (Figure 1). In the present review, we discuss the advantages of *Drosophila melanogaster* as a model organism by focusing, in particular, on PD and its related redox alterations, and emphasising the therapeutic potential of the antioxidant drugs. 

## 2. *Drosophila melanogaster* as a Model Organism in Scientific Research

The history of *Drosophila melanogaster* as a model organism in science began a little over 100 years ago, when Thomas Hunt Morgan and his school demonstrated the theory of chromosomal inheritance of Mendelian factors [10]. Over the years, *D. melanogaster* has become a model in modern genetics and is used for the study of several fundamental physiological and behavioural processes, most of which are conserved in higher eukaryotes, including mammals. In addition, *D*. *melanogaster* is considered a valuable model with which to investigate different aspects of human pathologies in translational studies.

Several factors have contributed to make *D*. *melanogaster* an informative model. Some are linked to the intrinsic characteristics of the organism, such as its short life cycle (about 10 days at 25 °C), high fecundity (females lay more than 800 eggs during their lifetime), high number of progeny per generation, and the absence of meiotic recombination in males. Furthermore, fruit flies are easy to grow and manipulate in the laboratory, and the generation of fly mutant strains has become relatively easy (see below). All these aspects have facilitated genetic studies, including those requiring the generation of specific fly lines or high numbers of individuals for powerful statistical analyses. 

Importantly, the *D. melanogaster* genome has been extensively studied, and it was completely sequenced in 2000 [11,12]. It encompasses ~143 Mbp, organised into four pairs of chromosomes—two X/Y sex chromosomes and three autosomes. The genome contains ~16,000 genes, of which ~13,000 code for proteins. Among the protein-coding genes, more than 60% have an orthologous counterpart in humans, representing promising candidates for translational studies [13]. In addition, for some cases in which human genes do not show an obvious fly homologue, fly–human conservation might still occur at the level of the pathway in which these factors are involved, making *Drosophila*-based studies again informative [13]. In the next section, we briefly describe the most common genetic tools used in fly studies.

### 2.1. Generation and Maintenance of Mutant Fly Lines 

Both forward and reverse genetic studies are based on the availability of mutant gene variants. Over the last century, several strategies and tools have been developed to obtain multiple collections of lines in *D. melanogaster*, characterised by different types of mutations. Mutant alleles are initially obtained using chemical (e.g., ethyl methanesulfonate, EMS) or radiation (X-ray) mutagenesis. The advent of the P-element-mediated transformation developed in flies by Rubin and Sprandling [14,15] raised the possibility of introducing any DNA sequence into the *Drosophila* genome, generating transgenic lines stably inheriting the inserted sequence. Methodologies based on fly transgenesis and random insertion of transposon vectors created large-scale collections of fly lines carrying mutant insertional alleles, including amorphic and hypomorphic variants [16,17]. Additional strategies exploiting homologous recombination (HR) [18] or the induction of double-strand breaks followed by HR, or error-prone nonhomologous end-joining such as TALENs and ZNFs [19,20], were established to target and modify specific genes. The possibility to mutate specific genes into the fly genome has been improved with the introduction of the CRISPR/Cas9 (clustered regularly interspaced short palindromic repeats/ CRISPR-associated nuclease) technology in *D. melanogaster*. Several groups contributed to the development of this methodology [21,22,23,24], making available to the fly community protocols and tools for using this strategy to generate mutant flies [25] (https://flycrispr.org/; https://www.crisprflydesign.org/). Recent advancements in CRISPR/Cas9 technology have allowed tissue-specific mutagenesis, which will permit fine dissection of the roles of specific genes in complex biological processes [26].

Independently of the strategy employed to obtain mutations, the availability of balancer chromosomes, peculiar chromosomes carrying multiple inversions, thereby inhibiting homologous recombination [27], allows the production of stock lines in which recessive lethal or sterile alleles can be stably maintained in heterozygosity. The use of balancer chromosomes also facilitates the production of fly lines carrying mutations at the level of multiple genes. 

### 2.2. The GAL4/UAS System

The possibility to manipulate gene expression and activity in a spatially and/or temporally controlled manner is one of the main strengths of the fly model. Common tools used for these analyses are based on the GAL4/UAS system originally developed by Brand and Perrimon [28]. In principle, this system relies on the generation of two different types of parental transgenic lines. The first parental line carries a construct in which a specific fly *cis*-regulatory sequence (enhancer and/or promoter region) promotes the expression of the coding region of the yeast transcription factor GAL4 (GAL4-driver). The second line carries a transgene in which DNA of interest is cloned under the control of the upstream activating sequences (UAS) specifically recognised by GAL4. In the progeny derived from the cross between the two parental lines, the UAS-controlled DNA sequence will be actively transcribed only in those tissues where GAL4 is expressed, allowing the analysis of its effect in a spatially-controlled way [28]. Moreover, GAL4 expression can be temporally regulated by adding to the system a third transgene specifying a temperature-sensitive transcription factor, GAL80^ts^. Indeed, GAL80^ts^ can bind to GAL4, repressing its transcriptional activity at the permissive temperature of 18 °C, while its inhibitory function is removed at higher temperatures, allowing GAL4 binding to UAS [29]. A recent refinement of the technique includes the split GAL4, which improves the temporal control of gene expression. In this system, the transcriptional activation domain of GAL4 and its DNA-binding domain are cloned in separated promoters. Hence, only when the two domains bind to each other is GAL4 reconstituted and functional [30]. 

The number of GAL4 and GAL80^ts^ lines has increased over time, allowing multiple and versatile combinations. Similarly, many transgenic UAS lines have been produced. Depending on the nature of the UAS construct, these lines can be used to study the effects of ectopic expression of a specific gene product, including those not present in the fly genome, or to explore the consequence of a gene’s downregulation via RNA interference. 

Other GAL4/UAS-based tools available for *Drosophila* are the EP lines. Each of these lines carries a randomly inserted EP element, a P transposon containing UAS sites. When activated using a GAL4-driver, the EP-element can induce mis-expression of endogenous genes located at the site of insertion. Such mis-expression can have phenotypic consequences, the characterisation of which allows the identification of novel genes or for new roles to be ascribed known genes. Thousands of EP lines have been produced and are available for screening studies [17,31,32,33]. 

### 2.3. Other Tools to Monitor Gene Expression in D. melanogaster

Different tools have been also developed to perform expression studies of fly proteins without the need for specific antibodies, which are often challenging to obtain for *Drosophila*. An early tool was based on the generation of transgenic lines carrying a randomly inserted green fluorescent protein (GFP)-coding artificial exon [34]. Subsequently, other methodologies, such as “MiMIC” (Minos-mediated integration cassette) [35] and “CRIMIC” (CRISPR mediated integration cassette) [36], based on the use of exchangeable ϕ-C3 integrase cassettes, were developed. In addition, strategies relying on the production of genomic P[acman] BACs or fosmids modified to tag protein-coding regions are also available [37,38,39]. These approaches allow characterisation of the expression of endogenous proteins at the tissue, cellular and subcellular levels as well as immunoprecipitation and live-imaging experiments, as reviewed in Reference [33]. Importantly, several lines for protein tagging studies are now available from public repositories. 

## 3. *D. melanogaster* as a Model to Investigate PD Pathology

The fly central nervous system is a bilaterally symmetrical brain composed of both neurons and glial cells. Notwithstanding its simplicity, the *Drosophila* brain coordinates complex behaviours such as circadian rhythms, sleep, memory, locomotion and learning. Moreover, *D. melanogaster* has been found to respond to drugs acting on the central nervous system similarly to mammals [40,41,42]. The fruit fly brain includes a well-characterised set of dopaminergic (DA) cells. DA neurons are subdivided into multiple clusters, which are symmetrically distributed and easily recognisable in the fly brain [43,44]. Similarly to mammals, the DA system is involved in the control of locomotion and other complex behaviours, including olfaction, memory, learning and sleep [45]. This remarkable degree of conservation, combined with the possibility of exposing flies to PD-linked toxins, has led to the investigation of PD-related phenotypes including DA neurodegeneration and dysfunctional locomotion. DA neuronal loss is normally evaluated by counting the number of neurons positive to tyrosine hydroxylase (TH) immunostaining, the rate-limiting enzyme in dopamine synthesis [44]. Locomotor alterations are usually evaluated by measuring startle-induced negative geotaxis, a typical *Drosophila* behaviour, also known as climbing. Once inside the experimental tubes, flies display the spontaneous tendency to climb following a slight stimulus [46]. Besides the availability of these tools, one great advantage of using *D. melanogaster* in the study of PD derives from the conservation of many PD-related genes. In fact, *parkin*, *Pink1*, *LRRK2* and *dj-1* mutant strains have been generated to investigate the mechanisms leading to the disease. Interestingly, *parkin* and *Pink1* mutants are characterised by reduced lifespan, mitochondrial pathology, apoptotic muscle degeneration and DA neuron degeneration [47,48]. Differently, *dj-1* loss of function confers milder phenotypes [49,50], which emerges particularly under oxidative treatments. Regarding dominant loci, *LRRK2*-null animals have been shown to present inconsistent phenotypes. Indeed, according to some reports, deficient flies show DA cell loss and locomotor deficits [51], while other studies did not find evidence of degeneration [52]. Interestingly, the expression of human *LRRK2* or the *Drosophila* homologue confers age-dependent DA cell death and locomotor alterations [53,54,55]. In contrast to the other PD genes, the fly genome does not bear an α-*synuclein* encoding gene. Hence, α-*synuclein* fly models are uniquely based on the ectopic expression of the human gene, both in mutated and wild-type form. Interestingly, transgenic flies overexpressing the protein develop LB-like inclusions, DA neuron degeneration and locomotor defects [56]. Furthermore, *Drosophila* shares high homology with the mammalian innate immune response of the brain, allowing the investigation of the PD-related neuroinflammatory mechanisms [57]. Therefore, considering these findings, the fruit fly represents a valuable system to model PD-linked genes and to dissect the related mechanisms of neurodegeneration. 

## 4. *Drosophila* as a Model to Evaluate In Vivo Redox Alterations 

Besides the conservation of several PD genes and the availability of behavioural tools, the potentiality of *Drosophila* as a PD model is further supported by the possibility of investigating in vivo the role played by oxidative stress in the neurodegenerative process. To date, several transgenic flies carrying redox-sensitive probes have been generated [58,59]. The existing sensors rely on redox-sensitive green fluorescent protein constructs (roGFP), which enable the exploration of cellular changes in the redox state mainly by exploiting glutathione oxidation (roGFP-Grx1, where roGFP is fused to glutaredoxin 1) or H_2_O_2_ concentration (roGFP-Orp1, where roGFP is linked to the H_2_O_2_-specific peroxidase Orp1) as readout [60]. Moreover, thank to further refinements, these probes can now be specifically expressed in different subcellular compartments, such as the cytosol or mitochondria, markedly improving the spatial resolution [59,61]. Transgenic fly lines harbouring these advanced sensors have been already used to explore the in vivo redox changes occurring in normal physiology and in pathological states [59,61]. For example, the use of the mitochondrially targeted H_2_O_2_-sensitive probes has shown that this ROS species is increased in both *Pink1* and *parkin* mutants, supporting the existence of a dysregulated redox homeostasis in these PD models [62]. Therefore, the availability of fly lines carrying redox-sensitive probes could significantly contribute to elucidation of the complex role played by the redox homeostasis in such pathological conditions.

**Figure 2:**
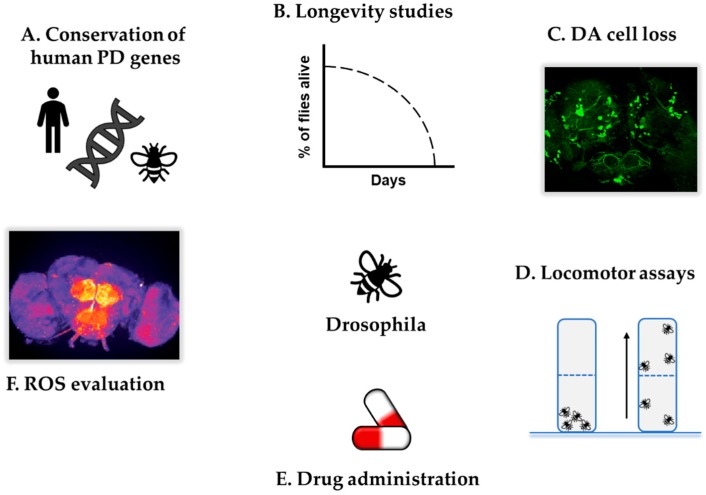
Advantages of *Drosophila melanogaster* as a model organism to investigate PD. *D. melanogaster* offers numerous advantages as a model organism for the study of PD-related features. (**A**) Fruit flies share many of the human genes involved in PD, allowing the generation of PD-mutant organisms which can then be investigated through different approaches; (**B**) longevity; (**C**) evaluation of dopaminergic (DA) cell loss; (**D**) locomotor defects, e.g., evaluated by climbing; (**E**) response to PD-toxins and therapeutic compounds and (**F**) ROS levels. The picture in (**C**) was produced in our laboratory by generating flies that overexpress GFP in DA neurons via *TH*-*GAL4* driver. The picture in (**F**) was adapted from Reference [62].

In addition to these useful tools, fruit flies have also been exploited to screen antioxidant compounds with therapeutic potential in neurodegenerative diseases. These molecules can be easily administered through the flies’ food and their effects evaluated over time through behavioural and biochemical analysis [63]. Ingestion can be actively monitored by adding inert coloured compounds to the standard fly food in which the molecule of interest is dissolved, or, alternatively, the compound solution can be directly injected into the fly abdomen to ensure complete absorption [63]. In this way, *Drosophila* can substitute for in vitro approaches, overcoming the limitations associated with cell-culture-based high-throughput screenings, which cannot mimic the whole-tissue response. Indeed, despite the introduction of 3D cultures, the results obtained with in vitro approaches cannot be generalised in vivo [64]. *Drosophila* represents a suitable in vivo model with which to perform preliminary drug screening studies to identify new candidate compounds [65,66], while also allowing the investigation of potential side effects, for instance by evaluating the mortality rate, embryonic development, adult morphology and behavioural traits [67,68]. Nonetheless, as drug response profiles can differ between fruit flies and humans due to the evolutionary distance, *Drosophila* should be considered as a preliminary drug discovery platform, the results of which should be further validated in higher model organisms to clarify their translational relevance. The major advantages of *D. melanogaster* as a model to study PD are summarised in Figure 2.

## 5. *Drosophila* as a Model to Probe Antioxidant Therapies in PD: Insights from *α-synuclein*, *parkin/Pink1* and *dj-1 PD* Models

In this section, we describe the use of *Drosophila* PD models that have mainly contributed to investigation of potential antioxidant drugs in the disease. Among the genetic models available, the ones that are mainly utilised are based on the heterologous expression of human α-*synuclein* or on the loss of function or downregulation of the fly homologues of *Parkin*, *PINK1* and *DJ-1*. Indeed, these fly lines are characterised by altered redox homeostasis, thus representing an appropriate model to evaluate antioxidant compounds. As summarised in Table 1, for each gene, we have provided a brief introduction followed by a description of its characterisation in *D. melanogaster* and the associated antioxidant-based studies.

### 5.1. α-synuclein Models

α-Synuclein is a small protein (14 kDa) particularly enriched at presynaptic terminals [92]. The protein is normally found in a dynamic equilibrium between a soluble cytosolic state and a membrane-bound state [92]. While in the former, the protein is unstructured, in the latter state, α-synuclein assumes an α-helix conformation. In its membrane-bound form, it seems to participate in neurotransmitter release and vesicle recycling, although the physiological function is still elusive [92]. Even though fruit flies lack a homologous gene, α-synuclein-overexpressing lines have been produced to investigate the physiopathological function of the protein. However, while the absence of a fly homologue allows specific investigation of the role of α-synuclein without any interference from an endogenous counterpart, in particular when pathological mutations are inserted in its sequence, the observed phenotypes could, on the other hand, represent a nonspecific consequence of foreign protein expression that cannot be properly managed by the organism. The overexpression of human α-synuclein and its A30P or A53T mutants does not affect the development of neuronal and non-neuronal tissues, nor does aging produce any gross, widespread degenerative changes [56]. However, flies expressing wild-type and mutant forms of the protein are characterised by a specific loss of dopaminergic neurons and by the appearance of α-synuclein aggregates that strongly resemble cortical LBs from patients with diffuse LB disease [56]. Together with these pathological phenotypes, the locomotion behaviour of *α-synuclein* transgenic flies has been demonstrated to decline more rapidly during aging than control flies, with a time course of climbing dysfunction that parallels the degeneration of dopaminergic neurons and the appearance of α-synuclein inclusions [56]. More recently, increased levels of ROS and cellular markers of oxidative damage such as lipid peroxidation and protein carbonylation have been measured in α-synuclein-expressing flies [69,70].

The presence of PD-related phenotypes in the α-synuclein-overexpressing flies has been used as a readout to test the protective effects mediated by endogenous detoxification pathways or by exogenous molecules. For instance, an impairment in glutathione metabolism has been shown to enhance the loss of dopaminergic neurons, whereas the overexpression of factors involved in glutathione biosynthesis and conjugation has been observed to suppress α-synuclein toxicity [71]. Interestingly, similar protective effects were also observed when feeding flies with sulphorafane and allyl disulfide, which have been demonstrated to significantly increase glutathione abundance [71]. In light of the known role of glutathione in protecting from oxidative stress, these data associate α-synuclein toxicity with oxidative damage. Accordingly, the overexpression of the enzyme methionine sulfoxide reductase A, which has been postulated to function as a catalytic antioxidant mechanism [72], has been demonstrated to markedly slow down the climbing deficits induced by wild-type or A30P α-synuclein and to preserve dopaminergic neurons from age-related degeneration [72]. In addition, statistically significant protection was observed when flies were fed with the non-toxic methionine analogue *S*-methyl-l-cysteine, which is a substrate of methionine sulfoxide reductase A [72]. Further experimental evidence on the involvement of oxidative damage in α-synuclein toxicity has arisen from a third study, in which both locomotion impairment and neuronal degeneration were reduced by the overexpression of superoxide dismutase 1 (SOD1), an antioxidant enzyme that participates in the removal of superoxide radicals [73]. In addition to the aforementioned studies aimed at evaluating how the activation of endogenous antioxidant pathways could be protective in the α-synuclein-based fly model of PD, more recent analyses have focused on the effects exerted by different natural antioxidant molecules, such as curcumin, epicatechin gallate, grape extracts, *Decalepis hamiltonii* root extracts and *Eucalyptus citriodora* and *Centella asiatica* leaf extracts [69,70,74,75,76,77]. Notably, every antioxidant molecule tested showed protective effects.

### 5.2. parkin and *PINK1* Models

As previously mentioned, *Parkin* and *PINK1* collaborate to drive the disposal of old or damaged mitochondria through a process called mitophagy [93]. Interestingly, studies carried out in *Drosophila* models first emphasised the role of both proteins in mitochondrial homeostasis [47,82,85]. When mitochondria are damaged, *PINK1* is stabilised on the mitochondrial outer membrane, where it recruits and phosphorylates parkin, inducing its E3 ligase activity, which labels mitochondria for degradation [93]. The phenotypes associated with the deletion of these genes have been largely characterised in *Drosophila* models, and most of them recapitulate the phenotypes observed in PD patients. Specifically, *parkin-*null mutants show a reduced life span and impaired locomotion activities [78,80,82,83]. Moreover, the degeneration of dopaminergic neurons has also been described, as well as mitochondrial defects [78,81]. The phenotypes associated with *Pink1* deficiency are almost identical to those observed in *parkin Drosophila* mutants [47,79,85]. Besides mitochondria alterations, *Pink1-* or *parkin*-null mutants have also been described to be associated with increased susceptibility to oxidative conditions. More specifically, *Pink1-*deficient flies show increased sensitivity to oxidative insults [85], while *parkin* mutant flies are characterised by an alteration in oxidative stress response [94] and enhanced sensitivity to oxygen radical injury [83]. More recently, through the use of redox-sensitive probes that specifically accumulate either at the cytosolic or mitochondrial level, we observed increased mitochondrial ROS in *parkin-* and *Pink1-*null mutants, while no significant changes in cytosolic ROS were detected [62].

The enhanced susceptibility to oxidative conditions has been the rationale for the analysis of the potential protective effects mediated by antioxidant molecules in *Pink1* and *parkin Drosophila* models of PD. First, as in the case of the α-synuclein model, alterations in glutathione metabolism have been shown to increase the neurodegenerative phenotypes of *parkin* mutants, while the overexpression of glutathione S-transferase S1 prevented dopaminergic neuron degeneration [81]. In another study performed in a knockdown *parkin* model, the pre-incubation of flies with a low concentration of paraquat (PQ) (0.1 mM) was shown to be able to prolong life-span and improve locomotion activity [84]. As PQ is a pesticide known to increase the production of superoxide radicals [95], these protective effects observed have been suggested to derive from an adaptive stress response referred to as “hormesis” [96]. In the same experimental model, when polyphenols such as propyl gallate or epigallocatechin gallate were added to low concentrations of PQ, a synergistic protective effect was observed. The antioxidant activity of those polyphenols was, however, unable to protect flies in the presence of higher PQ concentrations (>0.25 mM) [84]. In another study performed on a *Pink1* knockdown model, the expression of the human antioxidant enzyme SOD1 was shown to prevent the degeneration of dopaminergic neurons, confirming that the loss of *PINK1* is associated with increased oxidative conditions that can eventually lead to neuronal degeneration [86]. In light of the mitochondrial localisation of *PINK1*, and considering that mitochondria are the main endogenous source of ROS, PD-associated *Pink1* mutations might disrupt mitochondrial homeostasis, resulting in the production of radical species.

### 5.3. *DJ-1* Models

DJ-1 has been described as a multitasking protein principally localised in the cytoplasm [97]. Although its physiological function remains partially elusive, the protein has been suggested to participate in maintenance of the cellular redox state by regulation of the antioxidant defence [98]. Multiple pieces of evidence have shown that the absence of *DJ-1* results in impaired redox homeostasis, characterised by high levels of ROS [99]. Differently from humans, the *Drosophila* genome encodes two *DJ-1* homologues, referred to as *dj-1α* and *dj-1β*. While the expression of the protein *dj-1α* is restricted to the male testes, with a minor expression in the brain, *dj-1β* is ubiquitously distributed, similarly to the human protein. Double knockout flies are viable and fertile, though showing a higher sensitivity to oxidative insults [49,50]. Both factors have been shown to exert a protective function; however, *dj-1β* has been demonstrated to be primarily involved in the antioxidant protection due to its higher expression level and its ubiquitous pattern, which resembles the human protein [49]. In agreement with the proposed antioxidant role of *DJ-1*, higher levels of ROS and oxidative stress markers, such as protein carbonylation and lipid peroxidation, have been frequently observed in *dj-1*-deficient flies [87,88,100].

The sensitivity to oxidative stimuli of *dj-1*-deficient flies has been widely exploited in many studies, mostly utilising *dj-1β* null individuals as a platform for screening numerous antioxidants of both synthetic and natural origin. Among the natural molecules, the micronutrients vitamin C and vitamin E have been frequently used as antioxidants for their ability to buffer ROS and attenuate lipid peroxidation [101,102]. In this regard, a study conducted in *dj-1β* fly mutants demonstrated that the chronic administration of either vitamin C or vitamin E reduced protein carbonylation [88]. The same molecules were also assessed by another study for their ability to improve the longevity of *dj-1β-*null flies as compared to controls [87]. Interestingly, while vitamin C showed positive effects on both genotypes, vitamin E showed a pro-longevity effect only on the mutant line [87]. As *dj-1β*-null flies displayed higher levels of lipid peroxidation, the authors suggested that a vitamin-E-supplemented diet might be protective for the maintenance of membrane integrity in the absence of *dj-1β* [87]. Among the natural compounds, food supplementation with the cyanobacteria *Spirulina*, known for its antioxidant properties [103,104], has been shown to improve locomotion and survival rate of *dj-1β*-null flies under PQ exposure [90]. Moreover, epigallocatechin-3-gallate, a polyhydroxyphenol extracted from green tea [105], has been recently reported to positively affect the locomotor ability and survival rate of *dj-1β* knockout flies exposed to PQ [91]. Another natural molecule called Celastrol, derived from the root of a Chinese plant [89,106], has been described to rescue the dopaminergic neuronal loss and the dopamine content in flies silenced for the *dj-1α* homologue [89]. The same authors reported a similar protective activity for the semisynthetic tetracycline-derived antibiotic minocycline [89], which is emerging for its antioxidant and anti-inflammatory properties [107].

More recently, in an attempt to identify new drugs for PD treatment, Sanz and co-workers conducted a broad-spectrum drug screening study in *dj-1β*-null flies. Promising compounds were been selected according to the positive effect shown on climbing ability and on the levels of ROS and protein carbonylation of the mutant flies [65]. Besides the previously mentioned molecules, including minocycline and vitamin E, the authors also examined other candidate compounds, including pterostilbene, an extract of blueberries [108], methylene blue, a mitochondrial targeting antioxidant [109], dalfampridine, a potassium channel blocker used in multiple sclerosis treatment [110], sodium phenylbutyrate, a histone deacetylase inhibitor with multiple medical applications [111] and dexrazoxane, a cardioprotective drug [112]. Interestingly, all the aforementioned compounds have been shown to have antioxidative and protective properties [65]. As most of them have already been used in different medical applications, they appear to be promising candidates for further validation in antioxidant PD therapy.

## 6. Superoxide Radical Dismutation as a New Therapeutic Strategy in PD

As strongly emphasised in the previous sections, the notion that oxidative damage contributes to the neuronal degeneration observed in PD is commonly accepted today. However, in spite of the positive results obtained in many animal models of the disease, clinical trials aimed at evaluating the therapeutic potential of antioxidant drugs have been rather disappointing with mixed results, as extensively reviewed elsewhere [113]. Several reasons could account for the modest clinical outcomes, and a central issue seems related to the real nature of the radical species underlying the neuronal damage [5]. In other words, most of the tested antioxidant molecules targeted not the primary cause of the oxidative damage, but rather the downstream effects. Considering that mitochondria are the principal source of ROS and that superoxide anions are the primary radical species produced during the mitochondrial oxidative phosphorylation, it follows that a treatment strategy for oxidative stress that targets superoxide radicals could be more effective. Based on this rationale, we recently assessed the beneficial effects of antioxidant molecules capable of removing superoxide radicals in both sporadic and genetic fly models of PD [62,114].

As a proof of concept, we first tested the action of the endogenous superoxide dismutase enzymes SOD1 and SOD2 in flies treated with PQ or lacking either *PINK1* or parkin proteins. Both SOD1 and SOD2 were protective in our models, although with different effectiveness. More specifically, SOD1 exerted an elevated protective action in the presence of chronic PQ treatment, even when the protein was specifically overexpressed in dopaminergic neurons, but was ineffective with higher (acute) concentrations of PQ. In contrast, the protective effects of SOD2 were more evident in the presence of acute treatment [114]. The overexpression of SOD2, and to a lesser extent SOD1, was also able to ameliorate locomotion defects in *Pink1-* and *parkin*-null mutants [62]. Given the protective effects observed with both SOD1 and SOD2, in an important extension to the genetic manipulation of superoxide dismutation, we then explored the beneficial activity of the SOD-mimetic drug M40403. The choice of this molecule arose from its physicochemical properties and from the fact that it has already been evaluated in phase I and II clinical trials for the treatment of pain, which have indicated that it is safe and well-tolerated [115]. M40403 is a stable Mn(II) complex which is excreted intact with no detectable dissociation when intravenously injected in rats [116]. Moreover, the molecule is water-soluble and it is able to cross the blood–brain barrier, a mandatory property for a drug to be effective in PD [116]. Another important feature of M40403 is its ability to dismutate superoxide anions in a catalytic way, with a catalytic rate comparable to that of native SOD2 enzymatic activity or an order of magnitude lower, depending on the pH of the solution [116]. When tested in our PQ-based *Drosophila* models, M40403 was able to rescue the lethality induced by elevated concentrations of PQ and improve the locomotion behaviours of flies treated with sub-lethal concentrations of PQ [114]. Beneficial effects were also observed from the systemic administration of M40403 in both *Pink1-* and *parkin*-null mutants, even though the dose–response effects were slightly different between the two fly models [62]. Overall, our results indicate that the selective removal of superoxide anions could represent a new and more effective approach to cope with the enhanced oxidative conditions associated with PD, and support the further exploration of exogenous SOD-related molecules as a therapeutic strategy against PD.

## 7. Conclusions

PD is a complex disorder in which genetic susceptibilities and environmental factors participate in the pathogenesis of the disease. The multifactorial origin and the numerous cellular pathways affected by the disorder make it difficult to define a therapeutic strategy able to hamper its progression, and the current pharmacological approach is mostly aimed at replacing the action of dopamine at the striatal level. The pathogenesis of PD has been largely studied in the last years and, even though it is still partially elusive, many factors have been clarified. Among them, the roles of mitochondrial dysfunction and oxidative damage have been clearly demonstrated and are commonly accepted. This aspect has supported testing of the protective actions of numerous natural or synthetic antioxidant molecules in different animal models of the disease. While every single model presents some of the phenotypes associated with the disease, none is able to reproduce every clinical and pathological feature of PD. This is most likely one of the reasons why the results observed in animal models are almost never reproduced in clinical trials. Among the different animal models available to test the protective action of antioxidant molecules, *D. melanogaster* presents several advantages. The rapid reproductive cycle, short lifespan, powerful genetic tools and exemption from restrictive animal monitoring regulations allow for the rapid exploration of the proposed hypotheses in vivo on a statistically powerful number of individuals. Moreover, the genome encodes homologues for most of the currently identified PD genes [117], making it possible to easily evaluate a purported drug in different models of the disorder. One of the key aspects to be considered in the definition of successful antioxidant-based therapies is the nature of the oxidative species involved in the pathology. Given that the accumulation of dysfunctional mitochondria, which inevitably produce high levels of superoxide anions, is a key pathological factor associated with PD, it follows that molecules specifically directed against this radical species should be more effective in comparison to compounds that target the downstream effects of superoxide radical accumulation. While further work is required for a better understanding of the detrimental effects induced by the accumulation of superoxide radicals on the neuronal viability, the exploration of exogenous SOD-mimetic molecules as a therapeutic strategy against PD appears rather promising.

## Figures and Tables

**Table 1:** Antioxidant treatments positively evaluated in *Drosophila* genetic PD models.

Fly PD Model	Associated PD Phenotypes	Antioxidant Molecules/Pathways Tested
***α-synuclein*** (overexpression of the human homologue)	LB-like inclusions, DA degeneration, climbing defects [56], lipid peroxidation and protein carbonylation [69,70]	GstS1 upregulation [71] and overexpression of bovine MSRA [72] or human SOD1 [73] *S*-methyl-l-cysteine [72], curcumin [74], epicatechin gallate [75], grape extract [76], *Decalepis hamiltonii* [70], *Eucalyptus citriodora* [77] and *Centella asiatica* [69]
***parkin*** (Loss of function)	DA degeneration [78,79,80,81], climbing defects [79,81,82], mitochondrial dysfunctions [79,80,83] and sensitivity to oxidative treatments [83]	GstS1 upregulation [81] and overexpression of fly Sod1 and Sod2 [62] polyphenols (propyl gallate and epigallocatechin gallate) [84] M40403 (SOD-mimetic) [62]
***pink1*** (Loss of function)	DA degeneration [47,79], climbing defects [47,79], mitochondrial dysfunctions [47,79,85] and sensitivity to oxidative treatments [85]	overexpression of human SOD1 [86] and of fly Sod1 and Sod2 [62] M40403 (SOD-mimetic) [62]
***dj-1α and *dj-1β**** (Loss of function, single and double mutants)	Sensitivity to oxidative treatments [49,50], lipid peroxidation and protein carbonylation [87,88]	minocycline and celastrol [89] vitamin C and vitamin E [87,88], Spirulina [90], epigallocatechin-3-gallate [91], pterostilbene, methylene blue, dalfampridine, sodium phenylbutyrate and dexrazoxane [65]

DA: dopaminergic neurons; GstS1: glutathione-S-transferase S1; LBs: Lewy bodies; MSRA: methionine sulfoxide reductase A; SOD1: superoxide dismutase 1.

## References

[1] Przedborski S. (2017). The two-century journey of Parkinson disease research. Nat. Rev. Neurosci..

[2] Dickson D.W. (2012). Parkinson’s disease and parkinsonism: Neuropathology. Cold Spring Harb. Perspect. Med..

[3] Karimi-Moghadam A., Charsouei S., Bell B., Jabalameli M.R. (2018). Parkinson Disease from Mendelian Forms to Genetic Susceptibility: New Molecular Insights into the Neurodegeneration Process. Cell. Mol. Neurobiol..

[4] Kalia L.V., Lang A.E. (2015). Parkinson’s disease. Lancet.

[5] De Lazzari F., Bubacco L., Whitworth A.J., Bisaglia M. (2018). Superoxide Radical Dismutation as New Therapeutic Strategy in Parkinson’s Disease. Aging Dis..

[6] Haddad D., Nakamura K. (2015). Understanding the susceptibility of dopamine neurons to mitochondrial stressors in Parkinson’s disease. FEBS Lett..

[7] Chen C., Turnbull D.M., Reeve A.K. (2019). Mitochondrial Dysfunction in Parkinson’s Disease-Cause or Consequence?. Biology.

[8] Gelders G., Baekelandt V., Van der Perren A. (2018). Linking Neuroinflammation and Neurodegeneration in Parkinson’s Disease. J. Immunol. Res..

[9] Bisaglia M., Filograna R., Beltramini M., Bubacco L. (2014). Are dopamine derivatives implicated in the pathogenesis of Parkinson’s disease?. Ageing Res. Rev..

[10] Morgan T.H. (1915). The mechanism of Mendelian Heredity.

[11] Adams M.D., Celniker S.E., Holt R.A., Evans C.A., Gocayne J.D., Amanatides P.G., Scherer S.E., Li P.W., Hoskins R.A., Galle R.F. (2000). The genome sequence of Drosophila melanogaster. Science.

[12] Dos Santos G., Schroeder A.J., Goodman J.L., Strelets V.B., Crosby M.A., Thurmond J., Emmert D.B., Gelbart W.M. (2015). FlyBase: Introduction of the Drosophila melanogaster Release 6 reference genome assembly and large-scale migration of genome annotations. Nucleic Acids Res..

[13] Wangler M.F., Yamamoto S., Bellen H.J. (2015). Fruit flies in biomedical research. Genetics.

[14] Rubin G.M., Spradling A.C. (1982). Genetic transformation of Drosophila with transposable element vectors. Science.

[15] Spradling A.C., Rubin G.M. (1982). Transposition of cloned P elements into Drosophila germ line chromosomes. Science.

[16] Bellen H.J., Levis R.W., Liao G., He Y., Carlson J.W., Tsang G., Evans-Holm M., Hiesinger P.R., Schulze K.L., Rubin G.M. (2004). The BDGP gene disruption project: Single transposon insertions associated with 40% of Drosophila genes. Genetics.

[17] Bellen H.J., Levis R.W., He Y., Carlson J.W., Evans-Holm M., Bae E., Kim J., Metaxakis A., Savakis C., Schulze K.L. (2011). The Drosophila gene disruption project: Progress using transposons with distinctive site specificities. Genetics.

[18] Rong Y.S., Golic K.G. (2000). Gene targeting by homologous recombination in Drosophila. Science.

[19] Beumer K., Bhattacharyya G., Bibikova M., Trautman J.K., Carroll D. (2006). Efficient gene targeting in Drosophila with zinc-finger nucleases. Genetics.

[20] Liu J., Li C., Yu Z., Huang P., Wu H., Wei C., Zhu N., Shen Y., Chen Y., Zhang B. (2012). Efficient and specific modifications of the Drosophila genome by means of an easy TALEN strategy. J. Genet. Genom..

[21] Bassett A.R., Tibbit C., Ponting C.P., Liu J.L. (2013). Highly efficient targeted mutagenesis of Drosophila with the CRISPR/Cas9 system. Cell Rep..

[22] Yu Z., Ren M., Wang Z., Zhang B., Rong Y.S., Jiao R., Gao G. (2013). Highly efficient genome modifications mediated by CRISPR/Cas9 in Drosophila. Genetics.

[23] Gratz S.J., Cummings A.M., Nguyen J.N., Hamm D.C., Donohue L.K., Harrison M.M., Wildonger J., O’Connor-Giles K.M. (2013). Genome engineering of Drosophila with the CRISPR RNA-guided Cas9 nuclease. Genetics.

[24] Kondo S., Ueda R. (2013). Highly improved gene targeting by germline-specific Cas9 expression in Drosophila. Genetics.

[25] Gratz S.J., Rubinstein C.D., Harrison M.M., Wildonger J., O’Connor-Giles K.M. (2015). CRISPR-Cas9 Genome Editing in Drosophila. Curr. Protoc. Mol. Biol..

[26] Meltzer H., Marom E., Alyagor I., Mayseless O., Berkun V., Segal-Gilboa N., Unger T., Luginbuhl D., Schuldiner O. (2019). Tissue-specific (ts)CRISPR as an efficient strategy for in vivo screening in Drosophila. Nat. Commun..

[27] Muller H.J. (1927). Artificial Transmutation of the Gene. Science.

[28] Brand A.H., Perrimon N. (1993). Targeted gene expression as a means of altering cell fates and generating dominant phenotypes. Development.

[29] Zeidler M.P., Tan C., Bellaiche Y., Cherry S., Hader S., Gayko U., Perrimon N. (2004). Temperature-sensitive control of protein activity by conditionally splicing inteins. Nat. Biotechnol..

[30] Luan H., Peabody N.C., Vinson C.R., White B.H. (2006). Refined spatial manipulation of neuronal function by combinatorial restriction of transgene expression. Neuron.

[31] Rorth P. (1996). A modular misexpression screen in Drosophila detecting tissue-specific phenotypes. Proc. Natl. Acad. Sci. USA.

[32] Toba G., Ohsako T., Miyata N., Ohtsuka T., Seong K.H., Aigaki T. (1999). The gene search system. A method for efficient detection and rapid molecular identification of genes in Drosophila melanogaster. Genetics.

[33] Bilder D., Irvine K.D. (2017). Taking Stock of the Drosophila Research Ecosystem. Genetics.

[34] Morin X., Daneman R., Zavortink M., Chia W. (2001). A protein trap strategy to detect GFP-tagged proteins expressed from their endogenous loci in Drosophila. Proc. Natl. Acad. Sci. USA.

[35] Venken K.J., Schulze K.L., Haelterman N.A., Pan H., He Y., Evans-Holm M., Carlson J.W., Levis R.W., Spradling A.C., Hoskins R.A. (2011). MiMIC: A highly versatile transposon insertion resource for engineering Drosophila melanogaster genes. Nat. Methods.

[36] Lee P.T., Zirin J., Kanca O., Lin W.W., Schulze K.L., Li-Kroeger D., Tao R., Devereaux C., Hu Y., Chung V. (2018). A gene-specific T2A-GAL4 library for Drosophila. Elife.

[37] Ejsmont R.K., Sarov M., Winkler S., Lipinski K.A., Tomancak P. (2009). A toolkit for high-throughput, cross-species gene engineering in Drosophila. Nat. Methods.

[38] Venken K.J., Carlson J.W., Schulze K.L., Pan H., He Y., Spokony R., Wan K.H., Koriabine M., de Jong P.J., White K.P. (2009). Versatile P[acman] BAC libraries for transgenesis studies in Drosophila melanogaster. Nat. Methods.

[39] Sarov M., Barz C., Jambor H., Hein M.Y., Schmied C., Suchold D., Stender B., Janosch S., Vikas K.J.V., Krishnan R.T. (2016). A genome-wide resource for the analysis of protein localisation in Drosophila. Elife.

[40] Andretic R., Kim Y.C., Jones F.S., Han K.A., Greenspan R.J. (2008). Drosophila D1 dopamine receptor mediates caffeine-induced arousal. Proc. Natl. Acad. Sci. USA.

[41] Rothenfluh A., Heberlein U. (2002). Drugs, flies, and videotape: The effects of ethanol and cocaine on Drosophila locomotion. Curr. Opin. Neurobiol..

[42] Satta R., Dimitrijevic N., Manev H. (2003). Drosophila metabolize 1,4-butanediol into gamma-hydroxybutyric acid in vivo. Eur. J. Pharmacol..

[43] Whitworth A.J., Wes P.D., Pallanck L.J. (2006). Drosophila models pioneer a new approach to drug discovery for Parkinson’s disease. Drug Discov. Today.

[44] Mao Z., Davis R.L. (2009). Eight different types of dopaminergic neurons innervate the Drosophila mushroom body neuropil: Anatomical and physiological heterogeneity. Front. Neural. Circuits.

[45] Yamamoto S., Seto E.S. (2014). Dopamine dynamics and signaling in Drosophila: An overview of genes, drugs and behavioral paradigms. Exp. Anim..

[46] Nichols C.D., Becnel J., Pandey U.B. (2012). Methods to assay Drosophila behavior. J. Vis. Exp..

[47] Park J., Lee S.B., Lee S., Kim Y., Song S., Kim S., Bae E., Kim J., Shong M., Kim J.M. (2006). Mitochondrial dysfunction in Drosophila *PINK1* mutants is complemented by parkin. Nature.

[48] Pickrell A.M., Youle R.J. (2015). The roles of *PINK1*, parkin, and mitochondrial fidelity in Parkinson’s disease. Neuron.

[49] Meulener M., Whitworth A.J., Armstrong-Gold C.E., Rizzu P., Heutink P., Wes P.D., Pallanck L.J., Bonini N.M. (2005). Drosophila *DJ-1* mutants are selectively sensitive to environmental toxins associated with Parkinson’s disease. Curr. Biol..

[50] De Lazzari F., Bisaglia M., Zordan M.A., Sandrelli F. (2018). Circadian Rhythm Abnormalities in Parkinson’s Disease from Humans to Flies and Back. Int. J. Mol. Sci..

[51] Lee S.B., Kim W., Lee S., Chung J. (2007). Loss of *LRRK2*/PARK8 induces degeneration of dopaminergic neurons in Drosophila. Biochem. Biophys. Res. Commun..

[52] Wang D., Tang B., Zhao G., Pan Q., Xia K., Bodmer R., Zhang Z. (2008). Dispensable role of Drosophila ortholog of *LRRK2* kinase activity in survival of dopaminergic neurons. Mol. Neurodegener..

[53] Liu Z., Wang X., Yu Y., Li X., Wang T., Jiang H., Ren Q., Jiao Y., Sawa A., Moran T. (2008). A Drosophila model for *LRRK2*-linked parkinsonism. Proc. Natl. Acad. Sci. USA.

[54] Ng C.H., Mok S.Z., Koh C., Ouyang X., Fivaz M.L., Tan E.K., Dawson V.L., Dawson T.M., Yu F., Lim K.L. (2009). Parkin protects against *LRRK2* G2019S mutant-induced dopaminergic neurodegeneration in Drosophila. J. Neurosci..

[55] Venderova K., Kabbach G., Abdel-Messih E., Zhang Y., Parks R.J., Imai Y., Gehrke S., Ngsee J., Lavoie M.J., Slack R.S. (2009). Leucine-Rich Repeat Kinase 2 interacts with *Parkin*, *DJ-1* and PINK-1 in a Drosophila melanogaster model of Parkinson’s disease. Hum. Mol. Genet..

[56] Feany M.B., Bender W.W. (2000). A Drosophila model of Parkinson’s disease. Nature.

[57] Lye S.H., Chtarbanova S. (2018). Drosophila as a Model to Study Brain Innate Immunity in Health and Disease. Int. J. Mol. Sci..

[58] Liu Z., Celotto A.M., Romero G., Wipf P., Palladino M.J. (2012). Genetically encoded redox sensor identifies the role of ROS in degenerative and mitochondrial disease pathogenesis. Neurobiol. Dis..

[59] Albrecht S.C., Barata A.G., Grosshans J., Teleman A.A., Dick T.P. (2011). In vivo mapping of hydrogen peroxide and oxidized glutathione reveals chemical and regional specificity of redox homeostasis. Cell Metab..

[60] Ren W., Ai H.W. (2013). Genetically encoded fluorescent redox probes. Sensors.

[61] Stapper Z.A., Jahn T.R. (2018). Changes in Glutathione Redox Potential Are Linked to Abeta42-Induced Neurotoxicity. Cell Rep..

[62] Biosa A., Sanchez-Martinez A., Filograna R., Terriente-Felix A., Alam S.M., Beltramini M., Bubacco L., Bisaglia M., Whitworth A.J. (2018). Superoxide dismutating molecules rescue the toxic effects of *PINK1* and parkin loss. Hum. Mol. Genet..

[63] Pandey U.B., Nichols C.D. (2011). Human disease models in Drosophila melanogaster and the role of the fly in therapeutic drug discovery. Pharmacol. Rev..

[64] Langhans S.A. (2018). Three-Dimensional in Vitro Cell Culture Models in Drug Discovery and Drug Repositioning. Front. Pharmacol..

[65] Sanz F.J., Solana-Manrique C., Munoz-Soriano V., Calap-Quintana P., Molto M.D., Paricio N. (2017). Identification of potential therapeutic compounds for Parkinson’s disease using Drosophila and human cell models. Free Radic. Biol. Med..

[66] Maitra U., Ciesla L. (2019). Using Drosophila as a platform for drug discovery from natural products in Parkinson’s disease. Medchemcomm.

[67] Rand M.D. (2010). Drosophotoxicology: The growing potential for Drosophila in neurotoxicology. Neurotoxicol. Teratol..

[68] Papanikolopoulou K., Mudher A., Skoulakis E. (2019). An assessment of the translational relevance of Drosophila in drug discovery. Expert Opin. Drug Discov..

[69] Siddique Y.H., Naz F., Jyoti S., Fatima A., Khanam S., Rahul, Ali F., Mujtaba S.F., Faisal M. (2014). Effect of Centella asiatica Leaf Extract on the Dietary Supplementation in Transgenic Drosophila Model of Parkinson’s Disease. Parkinsons Dis..

[70] Jahromi S.R., Haddadi M., Shivanandappa T., Ramesh S.R. (2015). Attenuation of neuromotor deficits by natural antioxidants of Decalepis hamiltonii in transgenic Drosophila model of Parkinson’s disease. Neuroscience.

[71] Trinh K., Moore K., Wes P.D., Muchowski P.J., Dey J., Andrews L., Pallanck L.J. (2008). Induction of the phase II detoxification pathway suppresses neuron loss in Drosophila models of Parkinson’s disease. J. Neurosci..

[72] Wassef R., Haenold R., Hansel A., Brot N., Heinemann S.H., Hoshi T. (2007). Methionine sulfoxide reductase A and a dietary supplement *S*-methyl-l-cysteine prevent Parkinson’s-like symptoms. J. Neurosci..

[73] Botella J.A., Bayersdorfer F., Schneuwly S. (2008). Superoxide dismutase overexpression protects dopaminergic neurons in a Drosophila model of Parkinson’s disease. Neurobiol. Dis..

[74] Siddique Y.H., Naz F., Jyoti S. (2014). Effect of curcumin on lifespan, activity pattern, oxidative stress, and apoptosis in the brains of transgenic Drosophila model of Parkinson’s disease. Biomed. Res. Int..

[75] Siddique Y.H., Jyoti S., Naz F. (2014). Effect of epicatechin gallate dietary supplementation on transgenic Drosophila model of Parkinson’s disease. J. Diet. Suppl..

[76] Long J., Gao H., Sun L., Liu J., Zhao-Wilson X. (2009). Grape extract protects mitochondria from oxidative damage and improves locomotor dysfunction and extends lifespan in a Drosophila Parkinson’s disease model. Rejuvenation Res..

[77] Siddique Y.H., Mujtaba S.F., Jyoti S., Naz F. (2013). GC-MS analysis of Eucalyptus citriodora leaf extract and its role on the dietary supplementation in transgenic Drosophila model of Parkinson’s disease. Food Chem. Toxicol..

[78] Sang T.K., Chang H.Y., Lawless G.M., Ratnaparkhi A., Mee L., Ackerson L.C., Maidment N.T., Krantz D.E., Jackson G.R. (2007). A Drosophila model of mutant human parkin-induced toxicity demonstrates selective loss of dopaminergic neurons and dependence on cellular dopamine. J. Neurosci..

[79] Tain L.S., Mortiboys H., Tao R.N., Ziviani E., Bandmann O., Whitworth A.J. (2009). Rapamycin activation of 4E-BP prevents parkinsonian dopaminergic neuron loss. Nat. Neurosci..

[80] Wang C., Lu R., Ouyang X., Ho M.W., Chia W., Yu F., Lim K.L. (2007). Drosophila overexpressing parkin R275W mutant exhibits dopaminergic neuron degeneration and mitochondrial abnormalities. J. Neurosci..

[81] Whitworth A.J., Theodore D.A., Greene J.C., Benes H., Wes P.D., Pallanck L.J. (2005). Increased glutathione S-transferase activity rescues dopaminergic neuron loss in a Drosophila model of Parkinson’s disease. Proc. Natl. Acad. Sci. USA.

[82] Greene J.C., Whitworth A.J., Kuo I., Andrews L.A., Feany M.B., Pallanck L.J. (2003). Mitochondrial pathology and apoptotic muscle degeneration in Drosophila parkin mutants. Proc. Natl. Acad. Sci. USA.

[83] Pesah Y., Pham T., Burgess H., Middlebrooks B., Verstreken P., Zhou Y., Harding M., Bellen H., Mardon G. (2004). Drosophila parkin mutants have decreased mass and cell size and increased sensitivity to oxygen radical stress. Development.

[84] Bonilla-Ramirez L., Jimenez-Del-Rio M., Velez-Pardo C. (2013). Low doses of paraquat and polyphenols prolong life span and locomotor activity in knock-down parkin Drosophila melanogaster exposed to oxidative stress stimuli: Implication in autosomal recessive juvenile parkinsonism. Gene.

[85] Clark I.E., Dodson M.W., Jiang C., Cao J.H., Huh J.R., Seol J.H., Yoo S.J., Hay B.A., Guo M. (2006). Drosophila *PINK1* is required for mitochondrial function and interacts genetically with parkin. Nature.

[86] Wang D., Qian L., Xiong H., Liu J., Neckameyer W.S., Oldham S., Xia K., Wang J., Bodmer R., Zhang Z. (2006). Antioxidants protect *PINK1*-dependent dopaminergic neurons in Drosophila. Proc. Natl. Acad. Sci. USA.

[87] Lavara-Culebras E., Munoz-Soriano V., Gomez-Pastor R., Matallana E., Paricio N. (2010). Effects of pharmacological agents on the lifespan phenotype of Drosophila *DJ-1*beta mutants. Gene.

[88] Casani S., Gomez-Pastor R., Matallana E., Paricio N. (2013). Antioxidant compound supplementation prevents oxidative damage in a Drosophila model of Parkinson’s disease. Free Radic. Biol. Med..

[89] Faust K., Gehrke S., Yang Y., Yang L., Beal M.F., Lu B. (2009). Neuroprotective effects of compounds with antioxidant and anti-inflammatory properties in a Drosophila model of Parkinson’s disease. BMC Neurosci..

[90] Kumar A., Christian P.K., Panchal K., Guruprasad B.R., Tiwari A.K. (2017). Supplementation of Spirulina (Arthrospira platensis) Improves Lifespan and Locomotor Activity in Paraquat-Sensitive *DJ-1*beta(Delta93) Flies, a Parkinson’s Disease Model in Drosophila melanogaster. J. Diet. Suppl..

[91] Martinez-Perez D.A., Jimenez-Del-Rio M., Velez-Pardo C. (2018). Epigallocatechin-3-Gallate Protects and Prevents Paraquat-Induced Oxidative Stress and Neurodegeneration in Knockdown *DJ-1*-beta Drosophila melanogaster. Neurotox Res..

[92] Burre J. (2015). The Synaptic Function of alpha-Synuclein. J. Parkinsons Dis..

[93] Whitworth A.J., Pallanck L.J. (2017). PINK1/Parkin mitophagy and neurodegeneration-what do we really know in vivo?. Curr. Opin. Genet. Dev..

[94] Greene J.C., Whitworth A.J., Andrews L.A., Parker T.J., Pallanck L.J. (2005). Genetic and genomic studies of Drosophila parkin mutants implicate oxidative stress and innate immune responses in pathogenesis. Hum. Mol. Genet..

[95] Franco R., Li S., Rodriguez-Rocha H., Burns M., Panayiotidis M.I. (2010). Molecular mechanisms of pesticide-induced neurotoxicity: Relevance to Parkinson’s disease. Chem. Biol. Interact..

[96] Mattson M.P. (2008). Hormesis defined. Ageing Res. Rev..

[97] Biosa A., Sandrelli F., Beltramini M., Greggio E., Bubacco L., Bisaglia M. (2017). Recent findings on the physiological function of *DJ-1*: Beyond Parkinson’s disease. Neurobiol. Dis..

[98] Raninga P.V., Di Trapani G., Tonissen K.F. (2017). The Multifaceted Roles of *DJ-1* as an Antioxidant. Adv. Exp. Med. Biol..

[99] Giaime E., Yamaguchi H., Gautier C.A., Kitada T., Shen J. (2012). Loss of *DJ-1* does not affect mitochondrial respiration but increases ROS production and mitochondrial permeability transition pore opening. PLoS ONE.

[100] Stefanatos R., Sriram A., Kiviranta E., Mohan A., Ayala V., Jacobs H.T., Pamplona R., Sanz A. (2012). dj-1beta regulates oxidative stress, insulin-like signaling and development in Drosophila melanogaster. Cell Cycle.

[101] Padayatty S.J., Katz A., Wang Y., Eck P., Kwon O., Lee J.H., Chen S., Corpe C., Dutta A., Dutta S.K. (2003). Vitamin C as an antioxidant: Evaluation of its role in disease prevention. J. Am. Coll. Nutr..

[102] Niki E. (2014). Role of vitamin E as a lipid-soluble peroxyl radical scavenger: In vitro and in vivo evidence. Free Radic. Biol. Med..

[103] Liu Q., Huang Y., Zhang R., Cai T., Cai Y. (2016). Medical Application of Spirulina platensis Derived C-Phycocyanin. Evid. Based Complement. Alternat. Med..

[104] Pleonsil P., Soogarun S., Suwanwong Y. (2013). Anti-oxidant activity of holo- and apo-c-phycocyanin and their protective effects on human erythrocytes. Int. J. Biol. Macromol..

[105] Lee L.S., Kim S.H., Kim Y.B., Kim Y.C. (2014). Quantitative analysis of major constituents in green tea with different plucking periods and their antioxidant activity. Molecules.

[106] Allison A.C., Cacabelos R., Lombardi V.R., Alvarez X.A., Vigo C. (2001). Celastrol, a potent antioxidant and anti-inflammatory drug, as a possible treatment for Alzheimer’s disease. Prog. Neuropsychopharmacol. Biol. Psychiatry.

[107] Cankaya S., Cankaya B., Kilic U., Kilic E., Yulug B. (2019). The therapeutic role of minocycline in Parkinson’s disease. Drugs Context.

[108] McCormack D., McFadden D. (2013). A review of pterostilbene antioxidant activity and disease modification. Oxid. Med. Cell. Longev..

[109] Tucker D., Lu Y., Zhang Q. (2018). From Mitochondrial Function to Neuroprotection-an Emerging Role for Methylene Blue. Mol. Neurobiol..

[110] Sahraian M.A., Maghzi A.H., Etemadifar M., Minagar A. (2011). Dalfampridine: Review of its efficacy in improving gait in patients with multiple sclerosis. J. Cent. Nerv. Syst. Dis..

[111] Iannitti T., Palmieri B. (2011). Clinical and experimental applications of sodium phenylbutyrate. Drugs R & D.

[112] Galetta F., Franzoni F., Cervetti G., Regoli F., Fallahi P., Tocchini L., Carpi A., Antonelli A., Petrini M., Santoro G. (2010). In vitro and in vivo study on the antioxidant activity of dexrazoxane. Biomed. Pharm..

[113] Filograna R., Beltramini M., Bubacco L., Bisaglia M. (2016). Anti-Oxidants in Parkinson’s Disease Therapy: A Critical Point of View. Curr. Neuropharmacol..

[114] Filograna R., Godena V.K., Sanchez-Martinez A., Ferrari E., Casella L., Beltramini M., Bubacco L., Whitworth A.J., Bisaglia M. (2016). Superoxide Dismutase (SOD)-mimetic M40403 Is Protective in Cell and Fly Models of Paraquat Toxicity: IMPLICATIONS FOR PARKINSON DISEASE. J. Biol. Chem..

[115] Murphy C.K., Fey E.G., Watkins B.A., Wong V., Rothstein D., Sonis S.T. (2008). Efficacy of superoxide dismutase mimetic M40403 in attenuating radiation-induced oral mucositis in hamsters. Clin. Cancer Res..

[116] Salvemini D., Wang Z.Q., Zweier J.L., Samouilov A., Macarthur H., Misko T.P., Currie M.G., Cuzzocrea S., Sikorski J.A., Riley D.P. (1999). A nonpeptidyl mimic of superoxide dismutase with therapeutic activity in rats. Science.

[117] Whitworth A.J. (2011). Drosophila models of Parkinson’s disease. Adv. Genet..

